# A Review of Technology-Enhanced Recreational Therapy in Mild Cognitive Impairment

**DOI:** 10.1093/geroni/igaf122.3872

**Published:** 2025-12-31

**Authors:** Hayun Back, Yongseop Kim, Rebecca Westenskow

**Affiliations:** University of Utah, Salt Lake City, Utah, United States; University of Utah, University of Utah, Utah, United States; University of Utah, Salt Lake City, Utah, United States

## Abstract

As the prevalence of mild cognitive impairment (MCI) and dementia continues to rise, there is growing interest in non-pharmacological interventions that promote cognitive and psychosocial well-being. Technology-enhanced recreational therapy (TERT) represents a promising approach that aligns with recreational therapy’s emphasis on activity-based cognitive rehabilitation. However, the scope, effectiveness, and limitations of TERT in supporting individuals with MCI and dementia remain insufficiently understood. This scoping review mapped current evidence on TERT interventions for older adults with MCI and dementia, identifying technologies, therapeutic goals, outcomes, and methodological gaps. According to Arksey and O’Malley’s framework and PRISMA guidelines, five databases were searched for peer-reviewed studies (2000–2024). Forty-three studies met inclusion criteria and were charted by technology type, population, goals, outcomes, and design. Immersive and mixed/unspecified VR dominated (n = 36), with few studies using mobile apps (n = 2), robots (n = 1), or multimodel platforms (n = 1). Populations included MCI (n = 11), dementia (n = 8), and broader aging groups (n = 16), often in long-term care (n = 16). Therapeutic aims clustered around cognition (n = 15), affect (n = 13), social engagement (n = 11), and reminiscence (n = 5). Most studies (31/43) reported positive outcomes in cognition; mixed findings (n = 12) reflected small samples and short interventions. Study designs included RCTs (n = 11) and reviews (n = 11), though many were preliminary. Common barriers included usability, cybersickness, and equipment costs. Evidence indicates TERT can improve cognitive stimulation, affect, and social connection for older adults with cognitive decline. However, methodological limitations and inequities in access restrict implementation. Future work should pursue rigorous, longitudinal trials and culturally responsive designs to ensure equitable translation into recreational therapy practice.

